# Annotation of G-Protein Coupled Receptors in the Genomes of Parasitic Blood Flukes

**DOI:** 10.17912/micropub.biology.000704

**Published:** 2023-01-11

**Authors:** Isaac K Kamara, Javit T Thao, Kirandeep Kaur, Nicolas J Wheeler, John D Chan

**Affiliations:** 1 University of Wisconsin - Oshkosh, Oshkosh, WI, USA; 2 University of Wisconsin - Eau Claire, Eau Claire, WI, USA

## Abstract

Infection with *Schistosoma* parasitic flatworms ( *Schistosoma haematobium, Schistosoma mansoni* and *Schistosoma japonicum* ) causes the neglected tropical disease schistosomiasis. There is a need to identify new chemotherapies to treat these parasites, and G-protein coupled receptors (GPCRs) are a logical druggable targets to explore given they control key aspects of schistosome biology such as neuromuscular function and reproduction. Updated chromosome level genome assemblies for each of the three major species have recently been released. However, studies on these GPCRs require accurate, updated genome annotations. Here, we have re-annotated the GPCRs present in each of the three major schistosome species.

**Figure 1. GPCRs of all three major schistosome species.**
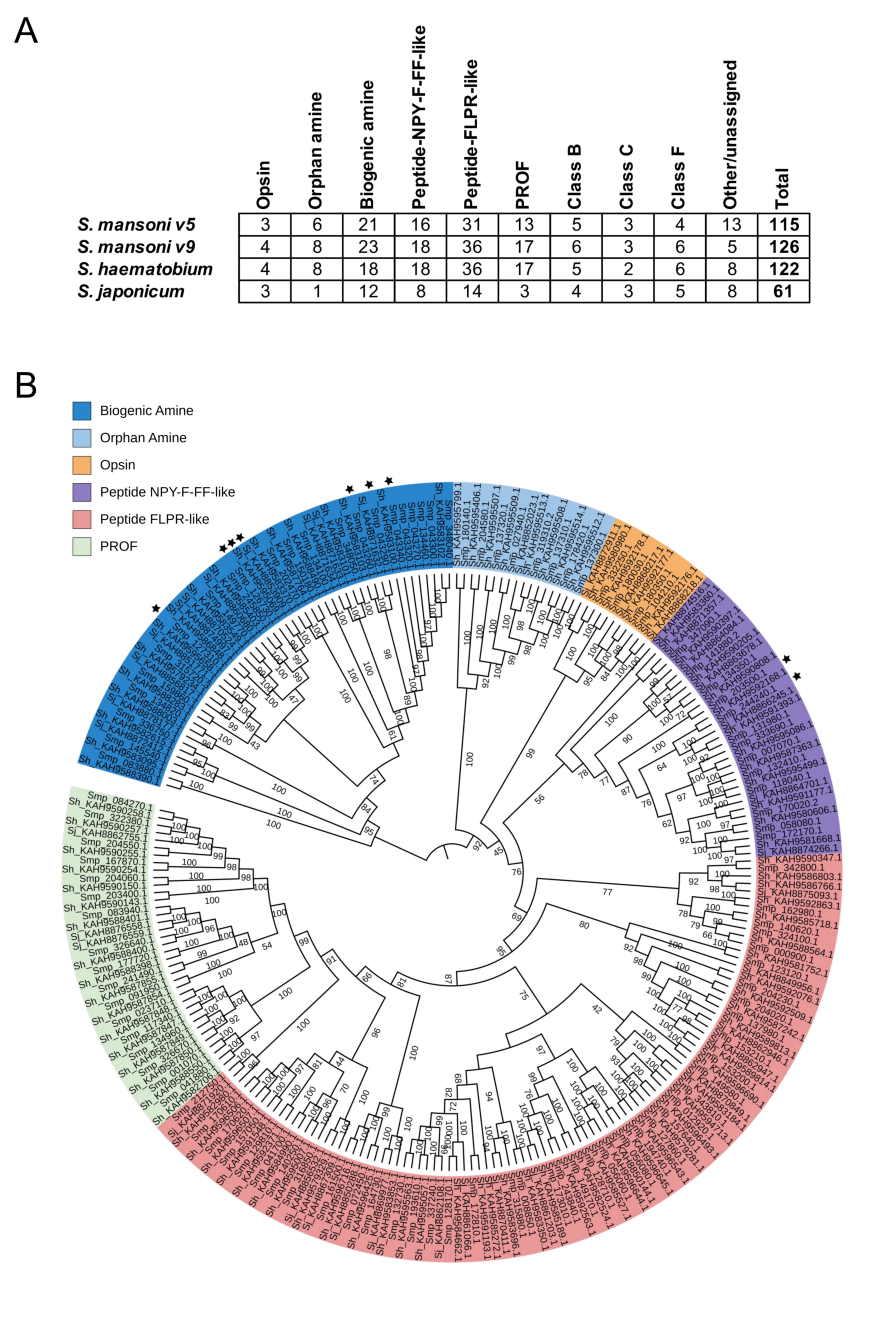
**A.** Number of GPCRs by class and subclass for all three major schistosome species. For *S. mansoni* , annotation of GPCRs identified in prior analysis of the v5 genome assembly (Hahnel et al., 2018) is compared to the annotation of the v9 genome in this study. **B.** Phylogenetic tree of schistosome Class A GPCRs. Sequences were aligned with MUSCLE and TrimAI was used to remove poorly aligned sequences and degap the alignment. IQ-TREE was then used to generate a maximum likelihood phylogeny (1000 bootstrap replicates). ★ = GPCR has been experimentally deorphanized.

## Description

Schistosomes are parasitic blood flukes that cause the neglected tropical disease schistosomiasis. Approximately 140 million people are estimated to be infected each year with the three major species causing human disease ( *S. haematobium* , *S. mansoni* and *S. japonicum* )(GBD 2019 Diseases and Injuries Collaborators, 2020). Expanded administration of praziquantel chemotherapy over the past decade has shown promising reductions in the prevalence of infections (Kokaliaris et al., 2022). However, since praziquantel monotherapy is the only treatment currently on the market, there is a need to identify additional antischistosomal targets and chemotherapies.

G-protein coupled receptors (GPCRs) are membrane receptors that signal through various second messengers or accessory proteins (ex. Gβγ or β-arrestin) to control a wide range of cellular processes. These receptors are involved in the mechanism of action for over 1/3rd of all FDA approved drugs (Hauser et al., 2017) and play a key role in numerous aspects of flatworm biology that could conceivably be subverted by anthelmintics. For example, GPCRs are involved in parasite neuromuscular function (reviewed in Ribeiro et al., 2012), reproduction (reviewed in Hahnel et al., 2018), and chemosensation (reviewed in Wheeler et al., 2022).

Several prior studies annotated the complement of GPCRs in different schistosomes as draft genomes for these species were released (Berriman et al., 2009; Campos et al., 2014; Hahnel et al., 2018; Zamanian et al., 2011). Recent advances in long read sequencing technologies have greatly improved genome assemblies from the initial versions published over a decade ago. Chromosome level assemblies have now been released for all three major species ( *S. mansoni* (Buddenborg et al., 2021), *S. haematobium* (Stroehlein et al., 2022) and *S. japonicum* (Luo et al., 2022)).

Updated annotations are necessary as these genome assemblies have become more complete and the gene models have improved. *S. mansoni* is the most commonly studied schistosome species in the laboratory and its genome assembly (PRJEA36577) has received the most efforts in iterative improvements over the years. The initial assembly reported 92 GPCRs (Berriman et al., 2009), and the most recent annotation effort counted 115 members of the family (Hahnel et al., 2018). However, that effort was working off of version 5 of the genome, and the current release of the *S. mansoni* chromosome level assembly is version 9 (Buddenborg et al., 2021). Similarly, annotation of GPCRs in the *S. heamatobium* genome utilized the initial draft genome published a decade ago (Campos et al., 2014; Young et al., 2012). Prior annotations have been valuable in facilitating experimental work on this gene family, but they likely represent incomplete estimates of the GPCRs present in these species.

We set out to generate a new phylogenetic analysis of GPCRs using the updated genome assemblies for *S. mansoni* (bioproject PRJEA36577, (Buddenborg et al., 2021)), *S. japonicum* (bioproject PRJNA739049, (Luo et al., 2022)) and *S. haematobium* (bioproject PRJNA78265, (Stroehlein et al., 2022)). Prior GPCR annotation studies have been published, and of these reference (Hahnel et al., 2018) is the most up to date and so protein sequences for this dataset (termed ‘ *S. mansoni* v5 GPCRs’) were used to search proteomes for all three species. Two approaches were taken to perform this initial search. First, *S. mansoni* v5 GPCRs were used as a query to perform a BLASTp search against the predicted proteomes for each of the new chromosome-level assemblies using a liberal cut off (E value < 10). Second, HMMER3 (v3.3.2) was used to search each of these proteomes using profiles built from *S. mansoni* v5 GPCRs annotated in (Hahnel et al., 2018).

These candidates were then triaged based on expected number of transmembrane domains (TMD). Topology prediction was performed with DeepTMHMM (Hallgren et al., 2022). While GPCRs have 7 TMD, sequences with between 4-10 TMD were retained to allow flexibility with potential errors in gene models. This yielded 183, 168 and 174 sequences for *S. mansoni, S. japonicum* and *S. haematobium* , respectively.

Finally, GPCR sequences were retained if they met one of the following two criteria. First, a BLASTp search was performed using sequences with 4-10 TMD as a query against the old *S. mansoni* v5 proteome. If the top hit for this backblast was annotated as a GPCR in (Hahnel et al., 2018) it was retained. Second, a conserved domain search was performed on the sequences with 4-10 TMD. Those sequences containing domains that correspond to GPCRs (for example, PFAM GPCR clan CL0192) were retained. The final count of *S. mansoni* GPCRs was 126, up from 115 reported in (Hahnel et al., 2018). However, the actual number of new *S. mansoni* GPCR sequences identified is 17. The reason for this is that several gene IDs in the *S. mansoni* v5 assembly used in (Hahnel et al., 2018) have been removed from the current *S. mansoni* assembly. *S. haematobium* contained 122 GPCRs, up from 79 reported in (Campos et al., 2014). *S. japonicum* contained 61 GPCRs, which is the first comprehensive GPCR annotation effort for this species.

These GPCR sequences were then classified as shown in Figure 1A. BLASTp search was performed to identify class B, C and F GPCRs, using the annotated *S. mansoni* v5 GPCRs as a query. This left the class A GPCRs, which were then used to produce the phylogenetic tree shown in Figure 1B. Briefly, amino acid sequences for class A GPCRs of all three species were aligned with MUSCLE, TrimAl (Capella-Gutiérrez et al., 2009) was used to identify poorly aligned sequences which were removed from the dataset, and IQ-TREE (Minh et al., 2020) was then used to generate a maximum likelihood phylogeny (Figure 1). This tree broadly reproduced the topology reported in (Hahnel et al., 2018). When the subclasses of GPCRs previously annotated for *S. mansoni* in (Hahnel et al., 2018) are considered (opsin, orphan amine, biogenic amine, peptide NPY-F-FF-like, peptide FLPR-like and PROF), we find that new GPCRs were added to each category (see the comparison of old v5 and current v9 GPCR count shown in Table 1). GPCRs that have been experimentally deorphanized are shown with stars on the phylogenetic tree. These are mainly restricted to the biogenic amines, where several receptors have been cloned and recombinantly expressed to validate ligand pairing. This includes a 5-HT7-like serotonin receptor (Chan et al., 2018, 2016; Patocka et al., 2014), a dopamine D2-like receptor (Taman and Ribeiro, 2009), a histamine-responsive receptor (El-Shehabi and Ribeiro, 2010) and a muscarinic acetylcholine receptor (MacDonald et al., 2015). Several Platyhelminth neuropeptide receptors have been functionally expressed ( (Omar et al., 2007; Saberi et al., 2016), , including two from *S. mansoni* (Weth et al. 2020), supporting the feasibility of flatworm peptidergic GPCR expression. The present dataset will hopefully facilitate further work in this area.

Differing GPCR numbers between species may be due to differences in methodology for genome sequencing, assembly and annotation, or they may reflect real biological differences between African and Asian schistosomes. The complement of *S. mansoni* and *S. haematobium* GPCRs are similar (both are African species infecting humans), while the number and distribution of GPCRs varies in *S. japonicum* (an Asian zoonotic species).

This dataset is intended as a resource to aid investigators studying schistosome GPCRs. However, a limitation is that these predictions are only as reliable as the annotated gene models. For genes that have very low expression in the biological samples being sequenced, there may be scarce transcriptomic data to guild *in silico* predictions. Conceivably, a GPCR could be important in a limited period during a life cycle stage underrepresented in sequencing data, and these gene models may not be predicted as accurately. It is possible that these annotations are still an incomplete count if there are partial gene models - although efforts were made to retain these sequences with a liberal cutoff of 4 TMD. Experiments on individual receptors will need to verify sequences (for example, by 5’/3’ RACE) to enable functional expression and receptor deorphanization. This resource is a starting point to enable those studies on GPCRs as potential anthelmintic drug targets.

## Methods

Predicted proteomes for the chomorosome level genome assemblies of the three species were retrieved as follows. *S. mansoni* protein sequences were downloaded from wormbase parasite (WBPS17), assembly SM_V9. For genes with multiple predicted transcripts, the longest representative was chosen and others were excluded from the dataset. *S. japonicum* protein sequences were downloaded from NCBI, bioproject accession number PRJNA739049, isolate jaV3_Hu on July, 2022. *S. haematobium* protein sequences for bioproject accession number PRJNA78265, Shae.V3 were downloaded from NCBI on July, 2022. Previously annotated *S. mansoni* v5 GPCR protein sequences were retrieved from the supplemental data in (Hahnel et al., 2018). BLASTp and HMMER (version 3.3.2) queries of these sequences against the predicted proteomes were performed locally. DeepTMHMM (version 1.0.18) was used for prediction of transmembrane topology, accessed online through https://dtu.biolib.com/DeepTMHMM/. Conserved domain search of candidate GPCRs was performed using Batch CD-Search (Lu et al., 2020) to search PFAM domains with an E value cuttoff of 0.01. Candidate GPCRs were considered to contain an annotated GPCR domains if they were found to have one of the following PFAM accession numbers or clan IDs; pfam00001, pfam00002, pfam13853, pfam10292, pfam10316, pfam10317, pfam10318, pfam10327, pfam10320, pfam10321, pfam10322, pfam10323, pfam10324, pfam10325, pfam10328, pfam01534, pfam11710, pfam11970, pfam02101, pfam03383, pfam03125, pfam02118, pfam02076, pfam05296, pfam03402, cl37946, cl28897, pfam01392, pfam00003, pfam01825. For phylogenetic analysis, multiple sequence alignment of the GPCR sequences was performed using MUSCLE and alignments were trimmed using TrimAl (Capella-Gutiérrez et al., 2009). Sequences that did not meet the criteria of 0.7 residue overlap and 70% sequence overlap were removed from the alignment, which was then realigned and trimmed in TrimAl with a 40% gap threshold. The resulting multiple-sequence alignment was then inputted into IQ-TREE (version 2.2.0; Minh et al., 2020), which constructed the maximum likelihood tree using standard model selection and 1000 bootstrap replicates. The phylogenetic tree was visualized using iTOL v6 (Letunic and Bork, 2021).

## Extended Data

Description: FASTA file of GPCR sequences shown in Figure 1. Resource Type: Dataset. DOI: 10.22002/19xvm-8d568
